# Could existing infrastructure for using patient‐reported outcomes as quality measures also be used for individual care in patients with colorectal cancer?

**DOI:** 10.1186/s12913-021-06457-6

**Published:** 2021-05-11

**Authors:** Clara Breidenbach, Christoph Kowalski, Simone Wesselmann, Nora Tabea Sibert

**Affiliations:** German Cancer Society, Kuno-Fischer-Straße 8, 14057 Berlin, Germany

**Keywords:** Patient‐reported outcomes, Patient‐reported outcome measures, Implementation, Integration, Routine care, EORTC, Inhibiting factors, Barriers, Facilitating factors, Facilitators

## Abstract

**Background:**

There has been increasing interest in integrating patient-reported outcomes (PROs) into routine oncological practice. To date, however, PROs have rarely been implemented in Germany. Currently, PROs are being used as performance measures in colorectal cancer centers in Germany. This content analysis identified factors that may inhibit or facilitate the additional use of PROMs for individual patient management.

**Methods:**

The analysis follows an exploratory approach. Out of 103 centers that participated in a multicentric PRO quality management and benchmarking program in Germany, twelve oncological health-care providers from eight certified colorectal cancer centers were interviewed using a semi-structured interview guide. The interviewees were clinicians (physicians, nurses, psycho-oncologist and physician assistant) who care for colorectal cancer patients. This analysis evaluated whether and how PROs that are primarily collected for quality management/benchmarking reasons could also be used for the management of individual patients. The data was analyzed using a content-analysis approach.

**Results:**

The interviewees were not using PRO in their routine clinical work, but they recognized its added value and pointed out potential example uses. Identified inhibiting factors for the use of PROs in clinical routine work were effortful access to PRO reports, lacking coordinating structures, time delays and time points of measurements as well as redundancy with other instruments. Facilitating factors for the use of PROs in clinical routine work that were identified included access via electronic patient records, implementation of coordinating structures for PRO processes in the center, clear PRO reports that are easy to interpret, and measurements at relevant time points.

**Discussion:**

Clinicians had quite a positive attitude toward PROs and recognized their added value. Inhibiting and facilitating factors of an organizational and technical nature were identified.

**Conclusions:**

These findings indicate how PROs used for quality management purposes may also be used for the management of individual patients. Therefore, existing structures and processes in the certified colorectal cancer centers, as well as lessons learned from the literature on the implementation of PROs monitoring individual patients need to be taken into account.

**Supplementary Information:**

The online version contains supplementary material available at 10.1186/s12913-021-06457-6.

## Introduction

Patient-reported outcomes (PROs) appear to have a strong potential to contribute to patient-centered oncological care [1–3]. PROs have been shown to facilitate interprofessional communications, as well as patient–clinician communications, and to support clinicians in their assessment of oncological patients’ symptoms by detecting underestimated side effects [4, 5]. PRO monitoring has also been associated with an improved quality of life and longer survival times, as it assists with treatment planning and can call attention to early disease progression [6–9].

There has therefore been increasing interest in integrating PROs into routine oncological practice in order to improve clinical care [1]. To date, however, PROs are still rarely used in routine oncological care in Germany. Barriers and facilitators for clinicians’ usage of PRO have been identified to be of primarily technical and behavioral nature [10]. Identified barriers to implementing PROs include among other the clinicians’ belief of limited capacities to address PROs, limited resources in organizations to e.g. support PRO implementation administratively or PROs being not connected to existing workflow [1, 10, 11]. Examples for facilitators include choosing PROs that are relevant to clinicians, providing feedback to clinicians on patients’ scores on an easily accessible format and educating clinicians on PRO use [1, 2, 10, 11]. The graphical presentation of PRO has also been subject of interest in several investigations [12–14]. Recently, research on PRO implementation focuses on electronic real-time collection investigating the potentials and challenges of new technologies [15, 16].

Research on implementing PROs derives predominantly from the United Kingdom, the Netherlands, Canada, or the United States. In Germany, however, PRO implementation is confronted with particular challenges including a decentralized health care system and a high hospital density with a higher proportion of small hospitals. Furthermore, German hospitals are below those in the above mentioned countries in terms of digitalization [17]. This has the consequence that PROs are not implemented extensively yet and especially electronic based PROs are not as distributed as in other countries.

In Germany, the oncological care landscape is shaped by cancer centers that have received certification from the German Cancer Society. Approximately half of all newly diagnosed cancer patients are treated in certified cancer centers (248,929 primary cases treated in centers while the annual incidence of newly confirmed cancers is about 489,178) and about 45 % of all newly diagnosed colorectal cancer patients per year are treated in one of the certified colorectal cancer centers (Rückher et al., submitted). In order to receive certification, centers have to fulfill certain requirements, including structural characteristics such as the establishment of an interdisciplinary tumor board or provision of psycho-oncological and social counseling services, or the introduction of specific personnel posts such as a center coordinator. Whether the center meets these requirements is monitored annually both on the basis of data submitted and through on-site auditing. With a current total of around 290 centers and nationwide distribution, certified colorectal cancer centers represent major standardized structures for oncological care that are currently available in Germany.

In view of the favorable effects of PROs, efforts have been made to integrate them into routine care. One example of this is the EDIUM study (acronym for German translation of “Outcomes in Colorectal Cancer: Identification of Differences and Measures for Nationwide Quality Development”^1^). Its primary objective is to compare PROs from certified colorectal cancer centers in Germany and identify potential variation for quality management [18]. It was intended that after finalizing the study and having integrated an infrastructure for PROs, centers could continue with their PRO collection. And in fact, after finalizing the data collection phase of EDIUM, almost half of the initial study centers continued to collect PROs routinely. Though PROs in the study are primarily used to allow for comparison across centers and quality management they may also be used for clinical decision-making or monitoring of individual patients since individual PROs per patient are provided to the clinicians.

Although existing research has documented barriers to and facilitators for PRO implementation [1, 10, 11], reviews show that little attention has been given to the specific contexts in which PROs are used [11, 19]. The objective of the analysis is therefore to identify factors that inhibit or facilitate the usage of PROs for clinical decision-making and monitoring patients in existing structures for oncological care, certified colorectal cancer centers in Germany. Regarding theoretical relevance, this analysis may contribute to knowledge about how specific contexts may change barriers and facilitators for PRO implementation. In terms of practical relevance, this analysis may indicate how PROs that are already integrated in routine care for quality management can be integrated for monitoring individual patients and individual decision-making. It might provide an overview of PRO implementation challenges specific to Germany and to certified cancer centers which constitute highly relevant oncological structures in Germany.

## Methods

### Study design and data collection procedures

EDIUM is a multicenter, prospective observational study that firstly aims at comparing PROs across colorectal cancer centers. 103 centers participate in the EDIUM-study. The patients included in the study complete questionnaires on quality of life — the European Organization for Research and Treatment of Cancer (EORTC) QLQ-C30 and EORTC QLQ-CR29 — at two time points: at the baseline before starting treatment, and 12 months after the start of treatment. The patients can complete the questionnaires either online or on paper, at home or in the center. Most of the questionnaires are filled out on paper. The individual patient responses are reported either by mail or via a web application to the colorectal cancer center that is treating them, and the team treating them has an opportunity to examine the individual patient responses. Concerning responses are not highlighted. A manual with reference values [20, 21] on how to read individual patient responses was provided to the colorectal cancer centers. Reporting to the colorectal cancer center is immediate if a questionnaire is completed online. If the questionnaire is filled in on paper, it is transferred to the central study office (pseudonymized), scanned, and reported to the colorectal cancer center after scanning and quality assurance.

The current analysis targets EDIUM’s second objective of evaluating whether the given PRO procedure is suitable to individual decision-making and monitoring patients and follows an exploratory approach. Data was collected by means of interviews held in November and December 2019, using a semi-structured interview guide. At the time of the interviews, baseline data collection of PROs for quality measurement had been taking place for approximately 11 months. The first part of the interview guide was designed to explore the providers’ attitude toward PROs, their experience with collecting and using or not using PROs in the EDIUM study, and their preferences regarding access to PROs (see Additional File 1 for the interview guide). The interview guide was developed upon insights the interviewers gained from communication to the colorectal cancer centers in their function as research assistants in the EDIUM team as well as prior research e.g. van Egdom et al. [10] including technical and behavioral issues such as IT, workflow and perceived value. In the second part of the interviews, the interviewers showed the interviewees examples of different presentation styles for PROs (bar chart, line graph, and tabular presentation as well as with and without colored-schemes/cut-off points; see Additional File 3). The presentation styles were supposed to serve as stimulation in order to discuss and evaluate the interviewees’ preferences regarding the presentation of PROs. Presentation styles as well as questions regarding the presentation styles were developed on the work of Brundage et al. [12] and Snyder et al. [13, 14] considering for example the identified preference for line-graphs, directionality issues, reference values and how to indicate possibly concerning results. Sociodemographic information on the interviewees regarding age, gender, and profession was also collected using a short questionnaire (see Additional File 1, part 3). The interview guide was evaluated by the interviewers after the first interview, but no changes were found to be necessary.

Twelve interviews were conducted (in one case, two interviewees participated together). The interviews lasted between 11 and 44 min. The two interviewers were female, are PhD candidates, are research assistants in the EDIUM study’s project management team, and are employees of the German Cancer Society, the institution that certifies the colorectal cancer centers. The interviewers attended several trainings regarding qualitative research methods including interview conducting and designing interview guides. The interviewers were known to the interviewees from the EDIUM team. The interviews were audio-recorded and then transcribed by the respective interviewer. The software program f4transkript, version 7.0.6, was used to transcribe the interviews. Few field notes were made during the interviews, however, they were not coded or analyzed. No repeat interviews were carried out and transcripts or findings were not returned to participants for comments/correction in order to minimize participants’ effort.

### Participant recruitment

The sampling technique was a mixture of convenience and snowball sampling. Each center participating in the EDIUM study (103 centers) had contact persons assigned for communications with the project’s management staff. In October 2019, these contact persons were invited via e-mail to nominate clinicians in their cancer center to take part in the interviews. The eligibility criteria for participation in the interviews was that the clinicians must be actively involved in providing care to colorectal cancer patients. Those who showed interest in being interviewed were contacted again in order to check eligibility criteria and arrange a meeting for the interview. One interested person could not be interviewed due to unmet eligibility criteria (did not work in care for patients with colorectal cancer). The interviews were conducted in person on the premises of the participating center. In one interview, the assistant of the interviewed chief physician was present, otherwise, only the interviewee(s) and the interviewer were present during an interview. Respondents provided written informed consent prior to participating in interviews.

Ethical approval was obtained by the Ethics Committees of the Berlin Chamber of Physicians (Eth-19/18). All participants gave written informed consent to participate in the study. Participants confidentiality was assured.

### Data analysis

The interviews were coded by two coders (who were the two interviewers) using a content analysis approach based on Kuckartz and McWhertor [22]. Initially, a few overall categories were built from the interview guide — e.g., “reasons for nonuse.” Additional categories and subunits were phrased inductively by paraphrasing, generalizing, and reducing quotations taken from the interviews.

As an initial step, both coders coded each interview independently. Both coders then met to jointly review and discuss unclear points and find consensus, as well as developed a code book containing the final list of codes (see Additional File 2). The software program f4analyse, version 2.5.4, was used to organize and manage the data.

## Results

### Sample characteristics

The final sample included different levels of professional experience, ranging from young professionals (e.g., assistant physicians) to people with extensive professional experience (e.g., chief physicians). Seven interviewees were physicians (five of them surgeons, and two were specialists for internal medicine). In addition, one interviewee was a psycho-oncologist, three interviewees were nurses (a stoma therapist and two nurses specializing in oncological care), and one interviewee was a physician assistant. Nine women and three men were interviewed. The interviewees’ ages ranged from 31 to 58 years, and their average age was 46. The sample included clinicians from eight different centers, three centers provided two to three interviewees. The centers were distributed across all regions in Germany.

### Actual PRO use and potential PRO use

The interviewed clinicians have access to the PROs for quality benchmarking at baseline and one year later. However, most of the interviewees stated that they had not used PROs in routine clinical work, either during the EDIUM study or in any other setting. Despite this reported nonuse of PRO reports for clinical decision-making, the clinicians who were interviewed expressed a positive attitude towards PROs and were able to identify potential example uses for them in routine clinical work. They thought that PROs could provide additional information about the patient that might be helpful in preparing for consultations with patients, could be used as a screening tool, might be helpful for treatment planning and for monitoring disease progression, and, last but not least, might be used to evaluate their own work. Potential example uses were identified by all of the interviewees – physicians, nurses and the psycho-oncologist.

“*I think it does actually give a very good additional view of the patients, because often not all of the topics included there can be fully covered in discussion during the medical consultation.” (specialist for internal medicine no. 1, paragraph 12)*.

*“Then for the next check-up, I’ll somehow know, ‘Ah, last time he was doing worse, now I’ll need to take a more careful look today.’ I think that’s more the way I’d see it.” (surgeon no. 2, paragraph 18)*.

*“If we weren’t seeing all of the patients personally, then I could imagine the questionnaire could also be quite useful for filtering out where we should go.” (psycho-oncologist, paragraph 16)*.

*“So, that I can go into the conversation and say [to the patient]: “You have filled out this questionnaire from the study. We have access to it, and I have seen that you have filled it out, so we should see what we can do with it.“ Somehow like that. Especially for consultation, either for inpatients, that you can already initiate something, or outpatient later. " (nurse no. 1, paragraph 22)*.

*“But the main thing I’m interested in is the questionnaire beforehand, because you can quickly find out from it lots of things you need to pay attention to, for treatment planning as well.” (specialist for internal medicine no. 1, paragraph 16)*.

*“And supposing these questionnaires become established now, maybe at each follow-up appointment, then I’d have a course like that and I could see whether it’s getting better or worse, and then it would actually help, I think.” (surgeon no. 2, paragraph 18)*.

*“You very rarely get this as a surgeon, because you never hear anything back from the patients, or when you ask about the follow-up. And so, if you can now practically trace the patient again, you’ve got a chance to say to yourself: it went well back then and also it’s still good. That’s also important, of course. Because you don’t get much positive feedback.” (surgeon no. 3, paragraph 20)*.

It was also mentioned that a single PRO questionnaire could be used instead of several different questionnaires — e.g., for the patient history and psycho-oncological screening — and that this could reduce unnecessary repetitions for patients and health professionals.

*“You don’t have to produce two questionnaires now if it’s possible just to use one for it, because the EDIUM questionnaire basically brings out everything almost as comprehensively as the thing we were thinking about using. And I think you can use it perfectly like this.” (surgeon no. 3, paragraph 16)*.

### Inhibiting and facilitating factors for the use of PROs in clinical routine work

The interviewees identified several technical and organizational factors that might influence whether PROs are used in clinical routine or not.

*Access.* It was repeatedly stated that the effort needed to access the patients’ responses to the questionnaire was a factor inhibiting the use of PROs. The interviewees said the PRO data had not yet been linked to the (electronic) patient records, meaning that a great deal of effort was needed to access the PROs.

*“It’s actually a relatively basic problem, it’s that you have to sit down and look through it again, log in again.” (surgeon no. 4, paragraph 18)*.

*“I’m just finding it difficult to access it at the moment, just for me personally. I think if it was a bit more transparent somehow it would definitely be easier, because you could link it somehow, but of course it’s all specific depending on the hospital and the state of the documents.” (*nurse no. 1, *paragraph 18)*.

In contrary, the interviewees repeatedly mentioned how important it was for PROs to be linked to the patients’ records. They believed this would enable clinicians, to easily review the PROs without any additional effort. In particular, it was emphasized that integrating them into the electronic patient records or electronic hospital information system would be relevant.

*“Well, I think the easiest thing to do in the end would be to integrate it into the kind of electronic “Fieberkurve”*^2^*that we’ll hopefully get sometime. So you could just say, OK — quality of life is one point you could click on and then you could see at a glance what the patient has ticked off. I think that’s really the only thing that makes sense. Because you could do that quickly during a handover, or when you’re checking lab values, you could take a quick look at it. And that’s it done with. So I think, I think that would be easiest. Because the questionnaire in the paper file is no use to anyone. You have to get hold of the file, it’s already far too time-consuming.” (surgeon no. 5, paragraph 26)*.

*Coordinating structures.* A lack of coordination between wards and among clinicians was given as one reason why PROs were not being used in the centers. As the interviewees saw it, this made it challenging to follow up on PRO monitoring. The psycho-oncologist, for example, stated that they receive little information about patients after they have received their surgery and have been relocated to other wards or to outpatient care. Another one said that only colleagues from one ward were aware of the fact that PROs were available, and that this information did not spread to other colleagues.

*“We probably won’t really hear about that here, those follow-up examinations. I mean, we do still sometimes attend to patients here during chemotherapy, but after that there’s nothing more.” (psycho-oncologist, paragraph 96)*.

*“But this way, it’s just me and a couple of colleagues or so, and we’ve also got the questionnaires, and for the rest of them it’s just more or less peripheral, they know the study is going on.” (surgeon no. 5, paragraph 18))*

This being said, it was suggested that a coordinating structure should be implemented for PRO processes. One interviewee proposed designating one person to be responsible for coordinating PRO monitoring in the center.

*“I think it’s just not established yet. I think if you had one person who was responsible for that alone, and — or if you said, OK, psycho-oncology can also use the questionnaire … I think that would mean a lot, it would work a lot better. Then we might also take a look at it.” (surgeon no. 5, paragraph 18)*.

*Time delays*. Time delays were also identified as an obstacle against using PROs. More than 93 % of the questionnaires are completed on paper in the EDIUM study, rather than online (interim study report to the centers) [23]. However, questionnaires that are completed on paper need to be sent to the central study office for scanning. The results of these questionnaires therefore arrive at the treating center after a time delay — typically after most treatment decisions have already been made and substantial parts of the treatment have already started.

*“And because it arrives after a certain amount of delay, most of the time it’s patients who have already just left. So it [the questionnaire] doesn’t actually influence my work that much at the moment.” (surgeon no. 2, paragraph 12)*.

*Time points.* In addition to this, it was also reported that the time point at which the measurements were made was a reason for not using PROs. Baseline measurements were perceived as irrelevant for clinical purposes in the present study, as they are mostly recorded shortly before treatment starts and the time for intervention is therefore too short. The intensity of patient contact was another reason for nonuse of baseline PRO reports. It was stated that during the baseline measurements, clinicians maintain close contact with the patients and therefore tend to rely more on their own clinical impressions than on the results of the questionnaire.

*“But to be quite honest, the time available is often also not enough. That would be the scale of it if the patients were to complete it as outpatients and we could already get it before they’re even admitted to the hospital. Would actually be helpful, but due to the DRG [Diagnosis Related Groups], the situation is usually that the patients arrive one day before the operation and then it’s really too late to intervene.” (surgeon no. 4, paragraph 24)*.

*“Because — just from our point of view now — before treatment, we have very close contact with the patients, we take our time to assess their condition and we think we can recognize the symptoms from a patient history and by seeing the patient during the rounds.” (surgeon no. 1, paragraph 16)*.

Some of the interviewees stated that the EORTC questionnaire used in the EDIUM study was redundant in addition to screening instruments that were already in use — e.g., screening for psycho-oncological requirements or patient histories.

*“No, it doesn’t have any advantages for me because I’ve got my own, I can show you it [shows the document]. We take a history for oncological care with the patients here, which we always go through with them. And some things in it are even the same as in this one [the EDIUM PRO report].” (nurse no. 2, paragraph 23)*.

The second time point for data collection, 1 year later, was perceived by surgeons as being too late, since clinicians on a surgical ward do not see patients that long after the operation, or because the patients’ problems might have been more relevant at an earlier time point.

*“Yes, I do think a year’s relatively long. And with procedures for colon cancer, it’s really, I think there are still current problems after 3 or 6 months, but they may possibly have declined again after a year.” (surgeon no. 4, paragraph 38)*.

Regarding facilitating factors, it was consistently stated that multiple assessments in the period between diagnosis and one year after the diagnosis would be important. There were differing opinions regarding relevant time points for PRO assessment. Surgeons repeatedly mentioned the initial weeks and also 3, 4, and 6 months after surgery as relevant time points.

*“Well, I think relatively soon after the operation would be quite good, so after three, four weeks - depending on the extent of the operation, such as recovery time, when one is perhaps back in everyday life to some extent.“ (surgeon no. 5, paragraph 28)*.

The specialists for internal medicine were also interested in long-term monitoring. One emphasized that assessments at later time points would also be important for monitoring quality of life in (long-term) survivors.

*“What would actually be nice would be to have a time point T2 and maybe T3. So I think it would be interesting to see how the patients get on afterward, you know? Especially the ones that aren’t then having palliative treatment. But those who have actually had surgery, which is a big majority of the patients.” (specialist for internal medicine no. 2, paragraph 95)*.

In addition, it was suggested from nurses, the psycho-oncologist and a surgeon that the assessments should be coordinated with events such as chemotherapy appointments, follow-up appointments, or rehabilitation activities.

*“And then after the first cycle, where everything’s usually still good. Exactly. And then simply after the third or fourth cycle, and maybe again toward the time after the sixth cycle, something like that so you could have different intervals in it, so you can observe some kind of course developing.” (nurse no. 1, paragraph 34)*.

*“And then just include it in the follow-up procedures, so that patients already know that when they get to the follow-up, the first thing that will happen is that they’ll get a questionnaire to fill out.” (surgeon no. 2, paragraph 52)*.

*"So especially during chemotherapy treatment, I could picture it." (psycho-oncologist, paragraph 30)*.

*PRO reports. *For PRO reports, most of the surgeons and nurses preferred charts (line graphs or bar charts) to a tabular presentation of the results. Reasons given for this included the view that charts were clearer and more intuitive than tabular presentations.

*“That’s what I grew up with.” (nurse no. 1, paragraph 44)*.

*“Because this, I think it’s the most concise way of doing it, so you can see it all at once. Ah, OK — preoperative, postoperative, postoperative. So then I can see it just like that, you know?” (nurse no. 3, paragraph 54)*.

One specialist for internal medicine and the psycho-oncologist did not have a strong opinion regarding the presentation styles. They thought that the tabular presentation was also clear.

*“Now for me […] subjectively [this] is actually the clearest [points to tabular presentation].“ (psycho-oncologist, paragraph 80)*.

On the one hand, surgeons and nurses mostly stated that a color scheme, like a traffic-light system, and cut-off points would be useful for interpreting the results easily during everyday routine work.

*“Because here you can see straight away: that’s red, that’s green, that’s yellow, that’s all more or less in the middle, and you can immediately see the course, I think that’s great.” (surgeon no. 5, paragraph 38)*.

*“Here I can see it quick as a flash, I mean the colors help a lot here. I mean, just to make it faster and more concise.” (nurse no. 3, paragraph 56)*.

On the other hand, the specialists for internal medicine also stated that using cut-off points might be misleading and should be applied well-considered.

*“They [reference values] shouldn’t suggest anything that isn’t meaningfully there. I mean, I think you have to be clear about what exactly you want to express with these reference values first. Otherwise, in the worst case, you’ll just be looking at the colors and thinking, ‘Oh, it’s all green,” without knowing what’s behind it. So I think, if you’re going to be using things like this, you should be able to spend a couple of minutes on them.” (*specialist for internal medicine no. 2, *paragraph 79)*.

In addition, it was consistently stated that all measurements for each patient should be shown in a single figure — e.g., the results at the first measurement point, the results at the second measurement point, and so on, so that PRO scores can be immediately compared.

*“Exactly, that shows a course then. That’s very important then.” (*specialists for internal medicine no. 1, *paragraph 28)*.

It was repeatedly pointed out that easy interpretation of PRO scores is important. A need for a clear thematic structure in the PRO scores was therefore noted.

*“But otherwise it’s already well structured, that you just have first the questions about general health and then disease-specific symptoms, from that point of view it’s already quite well done. And as I said, if you know that, then you can also use it quite well. It works quite well.” (*specialist for internal medicine no. 1, *paragraph 22)*.

The question arose of whether a report should list all of the scores or only abnormal results, in order to keep it short. The interviewees were almost unanimously in favor of listing all of the items in a report, to provide a comprehensive impression of the patient’s condition. Another suggestion was:

*“Or perhaps you could differentiate it and say you can make two buttons, with one showing only unusual features for a quick glimpse, or another where I can see the overall analysis of the questionnaire before and after for each patient. That would be great.” (surgeon no. 3, paragraph 46)*.

Finally, the interviewees reported difficulties in interpreting divergent directionalities in the PRO scores (EORTC). In the questionnaires used, higher values on some scores (function scores) indicated that the patient was feeling better, while on other scores (symptom scores) they meant the opposite. The interviewees thought the directionality of the interpretation should be consistent for all the PRO scores.

*“Yes, as I said, to start with it took quite a lot of getting used to, because you jump between functions and symptoms and then it goes back and forth a bit. You first have to get used to it.” (*specialist for internal medicine no. 1, *paragraph 22)*.

**Fig. 1:**
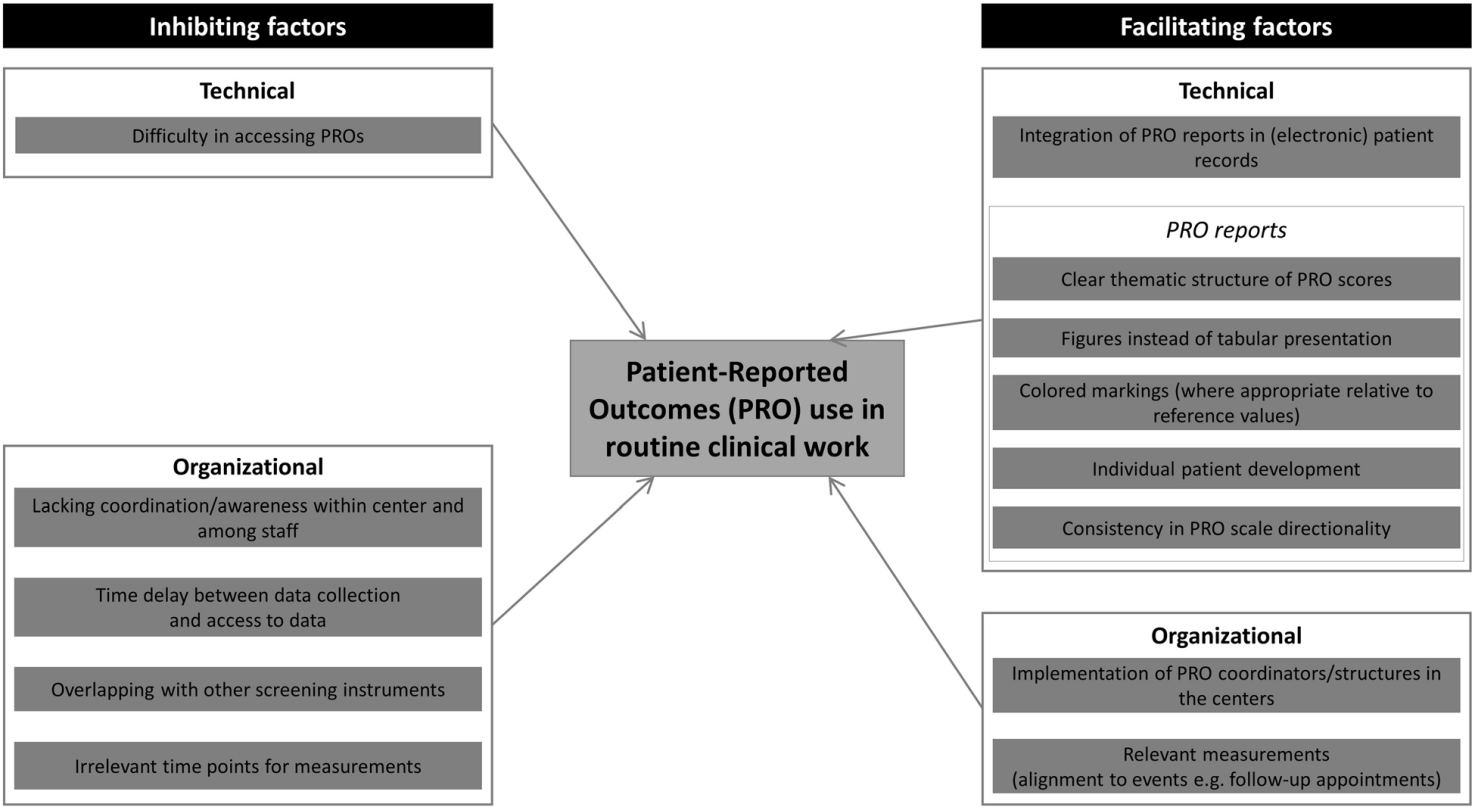
Factors identified as inhibiting or facilitating the use of patient-reported outcomes (PROs) in routine clinical work

Figure 1 summarizes the factors reported to be inhibiting or facilitating the use of PROs in routine clinical work in colorectal cancer centers.

## Discussion

The findings of this analysis add to the available information by identifying factors that inhibit or facilitate the usage of PROs in existing oncological structures. The present analysis focuses on the specific context of certified colorectal cancer centers in Germany and more specifically to an ongoing program that uses PROs primarily for quality management purposes but also allows for the use to assist management of individual patients.

The interviewees were not using PROs for routine clinical decision-making. However, they mostly reported positive attitudes towards PROs and recognized their added value. Potential example uses for PROs that they identified included providing additional information about the patient — e.g., in order to communicate with the patients — and serving as a screening instrument, for treatment planning and for disease monitoring, as well as for evaluating the clinicians’ own work. The research group has prepared an additional analysis in which this aspect is explored in more detail, to allow a deeper understanding of attitudes toward PROs among oncological staff [24]. This analysis reveals that interviewees had little knowledge about PROs in general.

Factors identified that inhibit or facilitate the use of PROs for clinical decision-making and individual patient monitoring included technical and organizational factors. With regard to technical factors, the findings underscore the importance of making PRO monitoring adaptable to and compatible with existing routine processes. In line with previous research, it was found that integrating PRO records into the electronic patient records might facilitate access to PRO reports and implementation of PRO monitoring in the everyday routine [11, 15, 16, 25]. With regard to the design of the PRO reports, the results show that clear presentation and ease of interpretation are extremely important for successful implementation. For clarity of presentation, figures are thought to be preferable to tabulated scores, scores should be structured thematically and consistency in scale directionality is important — findings that are in accordance with the results of a previous mixed-methods study and recommendations [12, 14]. In addition, reference values and highlighting of particular scores were regarded as controversial in this context. Especially surgeons regarded these as tools for easy interpretation, while specialists for internal medicine thought they might be misleading and that maintaining a holistic overview was important for appropriate clinical evaluation. A Delphi process including clinicians, patients and researchers identified a need for further research on the topic of reference values and labeling score ranges was needed [14].

It has been reported previously that designing and preparing processes and structures in advance for using PROs within an organization is crucial for successful implementation of PROs [11, 18, 25]. In the present study, the results also demonstrate a need for coordinating structures for PRO processes in the center, since the segmentation of care was perceived as impeding PRO monitoring. In the specific context of certified colorectal cancer centers, existing structures are available to which PRO processes might be attached. For example, a multidisciplinary team meets regularly at tumor boards in which the patients are discussed, and this might provide a suitable platform for integrating PRO reports. Another possible starting point could be to promote PRO processes through the certification requirements — e.g., implementing PRO structures that are then evaluated in the annual certification audits. In this way, quality management could further be used to include PROs as a parameter in clinical decision-making.

Previous reports in the literature have identified target group engagement as a relevant factor in overcoming barriers to PRO implementation in the provision of cancer care [18, 26]. In the present study, it emerged that it was also important whether or not the oncological staff perceived any benefit from PRO monitoring. It became apparent that the time point at which PRO assessments are made, as well as the parallel existence of other screening instruments, were related to the benefits perceived by the oncological staff. The time points for PRO assessment considered to be relevant differed between professions and disciplines, but they should be coordinated with follow-up appointments. Whereas surgeons preferred PRO assessment in the initial weeks after surgery, the specialists for internal medicine also highlighted the assessment of long-term PROs. This finding underlines Foster’s [11] point that the target group needs to be involved in the integration process right from the very start and that PRO applications should make it possible to collect PROs in a flexible manner. Moreover, a certain time delay of the PRO reports due to the mostly paper-based PRO collection was identified as inhibiting. Online PRO-collection should therefore be promoted in order to allow PRO reports be on time for follow-up appointments.

A few limitations to this analysis need to be mentioned. First of all, the results are based on a fairly small number of interviews, due to limited time or limited willingness to participate on the part of the eligible staff. Above that, no interviewees could be included that already routinely use PROs for clinical decision-making or patient monitoring since this was rare across the participating centers in EDIUM and Germany. However, it was possible to recruit a quite heterogeneous group of participants from different disciplines, age groups, levels of professional experience, gender, and geographical areas. Secondly, both interviewers are employees of the German Cancer Society, which is in charge of the certification system. The possibility can therefore not be excluded that this might have influenced the interviewees’ responses. However, it was made clear to all of the interviewees in advance that taking part in the interviews would have no impact on certification processes and that their confidentiality was assured.

## Conclusions

Certified cancer centers are a highly relevant structures in the German oncological landscape. The findings of this study may serve to improve infrastructure and procedures for the use of PROs for clinical decision-making in certified colorectal cancer centers in Germany and may also contribute to PRO implementation in other certified cancer centers in Germany (certified prostate cancer centers already collect PROs for quality management, as well). The presented results suggest that the infrastructure and procedures for using PROs for quality management reasons may require further adjustments for the usage of monitoring and decision-making of individual patients. The clinicians who were interviewed regarded PROs as providing added value for routine care and identified potential example uses. However, the implementation of PRO monitoring for clinical decision-making should be designed and planned while taking into consideration the unique systems and existing structures in the organization and in the target audiences e.g. that, in Germany, electronical PROs are at an earlier stage than in other countries. Identified barriers and facilitators were technical and organizational. Moreover, lessons learned from the literature on the implementation of PROs monitoring individual patients should be considered, such as international guidelines and recommendations of Crossnohere et al. [27]. The clinicians who are intended to use the PROs should be included in the implementation process right from the very start .

## Supplementary Information

**Additional file 1.** Interview Guide

**Additional file 2.** Code Book

**Additional file 3.** Presentation Styles PRO Reports

## Data Availability

According to the patient consent form data is not available for scientific use by others than the project group members (contact: Clara Breidenbach, breidenbach@krebsgesellschaft.de).

## Abbreviations

EORTC: European Organization for Research and Treatment of Cancer
PRO: patient-reported outcomes
DRG: Diagnosis Related Groups

## Footnotes

1 German: Ergebnisqualität bei Darmkrebs: Identifikation von Unterschieden und Maßnahmen zur flächendeckenden Qualitätsentwicklung.

2 German expression for a visualization of patients’ vital signs such as body temperature (German “Fieber” = fever), blood pressure etc.
