# Supplementary material for: Could existing infrastructure for using patient‐reported outcomes as quality measures also be used for individual care in patients with colorectal cancer?
Source: BMC Health Serv Res. 2021 May 11;21:448. doi: 10.1186/s12913-021-06457-6 (PMC8111716; doi:10.1186/s12913-021-06457-6)
Supplement: Supplementary file 1 — Additional file 1. Interview Guide [file 12913_2021_6457_MOESM1_ESM.docx]

Title: Could existing infrastructure for using patient-reported outcomes as quality measures in several health systems also be used for individual care in patients with colorectal cancer? Clinician perceptions of potential inhibiting and facilitating factors

**Authors**

1. Corresponding author:

Clara Breidenbach
German Cancer Society
Kuno-Fischer-Straße 8, 14057 Berlin, Germany
[breidenbach@krebsgesellschaft.de](mailto:breidenbach@krebsgesellschaft.de); +49 30 322 932 934

1. Christoph Kowalski
   German Cancer Society

Kuno-Fischer-Straße 8, 14057 Berlin, Germany
[kowalski@krebsgesellschaft.de](mailto:kowalski@krebsgesellschaft.de); +49 30 322 932 947

1. Simone Wesselmann
   German Cancer Society

Kuno-Fischer-Straße 8, 14057 Berlin, Germany
[wesselmann@krebsgesellschaft.de](mailto:wesselmann@krebsgesellschaft.de); +49 30 322 932 990

1. Nora Tabea Sibert

German Cancer Society
Kuno-Fischer-Straße 8, 14057 Berlin, Germany
[sibert@krebsgesellschaft.de](mailto:sibert@krebsgesellschaft.de); +49 30 322 932 968

# Additional File 1: Interview Guide

## Part 1: Main interview guide

| 1 To start with, could you briefly describe your work in the hospital? | - Field of activity/position - Ward - Working hours - How long have you had the position? |
| --- | --- |
| 2 How is the EDIUM study going for you?  — Or, referring to PROs more directly: how do you collect PROs in the framework of the EDIUM study? | - Patient information - Clarifying wording (what do you call PROs / quality-of-life profiles?) - Collecting PROs / quality-of-life data - Participants |
| 3 In what ways are you using the PROs / quality-of-life profiles clinically?  *Or:*  What sort of role do PROs play in your routine clinical work? | - Inclusion in treatment planning - Preparation for discussions - In which specialty? (Specialist service, psychological, oncological, nursing) - Importance in everyday clinical work - Reasons for use / nonuse   - Access options   - Presentation   - Importance / meaningfulness   - Embedding in workflows |
| 4 What do you think about the PROs? | - Benefits for patients - Benefits for clinical staff - Meaningfulness (general — e.g., scientific) - Presentation of PROs in EDIUM - Optional access in EDIUM |
| 5 If you could decide, how would PROs be used in your routine clinical work? | - Time point of survey - Process implementation (time point, participants, access) - Presentation |

## Part 2: show the interviewee three or four presentation options

| 1 Which aspects of the presentation are important for you?  How should PROs be presented so that they’re easy for you to work with? | - Form (paper or digital) - Conciseness / length - Clarity - Completeness (only relevant scores?) - Information content (individual questions) - Color design - Reference values - Interpretation aids (difference between function scale and symptom scale) |
| --- | --- |
| 2 What do you think of these presentation styles? | - When you look at these presentation styles for PROs, which ones would you prefer to use clinically? - Why this one? Why not the others? |
| 3 Do you have any other suggestions for improvement? Would you like to say / add anything else? Do you think there are any aspects that haven’t yet been covered? | |

*Part 3: Sociodemographic information*

1. In which year were you born?

2. What is your gender?

□ male

□ female

□ divers

3. Which profession do you have?

□ nurse

□ with specializing in oncology

□ without specializing in oncology

□ physician

□ assistant physician

□ specialist

□ senior physician

□ chief physician

□ psycho-oncologist

□ social worker

□ other: _________________________
