# Supplementary material for: Could existing infrastructure for using patient‐reported outcomes as quality measures also be used for individual care in patients with colorectal cancer?
Source: BMC Health Serv Res. 2021 May 11;21:448. doi: 10.1186/s12913-021-06457-6 (PMC8111716; doi:10.1186/s12913-021-06457-6)
Supplement: Supplementary file 2 — Additional file 2. Code Book [file 12913_2021_6457_MOESM2_ESM.docx]

Title: Could existing infrastructure for using patient-reported outcomes as quality measures in several health systems also be used for individual care in patients with colorectal cancer? Clinician perceptions of potential inhibiting and facilitating factors

Authors

1. Corresponding author:

Clara Breidenbach
German Cancer Society
Kuno-Fischer-Straße 8, 14057 Berlin, Germany
[breidenbach@krebsgesellschaft.de](mailto:breidenbach@krebsgesellschaft.de); +49 30 322 932 934

1. Christoph Kowalski
   German Cancer Society

Kuno-Fischer-Straße 8, 14057 Berlin, Germany
[kowalski@krebsgesellschaft.de](mailto:kowalski@krebsgesellschaft.de); +49 30 322 932 947

1. Simone Wesselmann
   German Cancer Society

Kuno-Fischer-Straße 8, 14057 Berlin, Germany
[wesselmann@krebsgesellschaft.de](mailto:wesselmann@krebsgesellschaft.de); +49 30 322 932 990

1. Nora Tabea Sibert

German Cancer Society
Kuno-Fischer-Straße 8, 14057 Berlin, Germany
[sibert@krebsgesellschaft.de](mailto:sibert@krebsgesellschaft.de); +49 30 322 932 968

**Additional File 2: Code Book**

| **Category/subunits** | | | | **Definition/rule** | **Example** |
| --- | --- | --- | --- | --- | --- |
| **1. Clinical usage** | | | | Category that structures subunits; built from interview guide | Quotes are assigned to smallest subunits in this category |
|  | **1.1. Nonuse** | | | Category that structures subunits;  built from interview guide |  |
|  | | **1.1.2. Barriers** | | Built from interview guide.  Contains subunits with information about why PROs are currently not used. |  |
|  | | | **Lack of coordination in the center** | Built inductively from the data.  If interviewee states that there is a lack of personnel structures relative to PRO usage. | “And then right after that — usually at six in the evening — you explain the procedure to the patient, who then has the operation the next day, and sometimes you never see them again because they’ve moved to a different ward. That’s another thing that makes direct links difficult.” (surgeon no. 5, paragraph 16) |
|  | | | **Access problems** | Built inductively from the data.  If interviewee states that there are problems with accessing the PROs. | “I’m just finding it difficult to access it at the moment, just for me personally. I think if it was a bit more transparent somehow it would definitely be easier, because you could link it somehow, but of course it’s all specific depending on the hospital and the state of the documents.” (nurse no. 1, paragraph 18) |
|  | | | **Time delay** | Built inductively from the data.  If interviewee states that when the questionnaire is filled out on paper, there is a delay in receiving the results. | “Especially since they arrive with a certain amount of delay — but I think that’s our fault, you know? I mean, I think it’s because XY only reports it to you with a delay, because I think she then has to compare it with the OncoBox and that’s in a separate place here with us. And she doesn’t have any access to the data from the OncoBox, so it has to be compared first. And because it arrives after a certain amount of delay, most of the time it’s patients who have already just left. So it doesn’t actually influence my work that much at the moment.” (surgeon no. 2, paragraph 12) |
|  | | | **Time for intervention too short** | Built inductively from the data.  If interviewee states that the time point for the baseline measurement is too close to the start of treatment, which is why it is not relevant for clinical decision-making. | “Well, I don’t think you’re really going to go into that [before treatment]. Because time’s also very short, if we can put it like that, because you have to do it before treatment.” (specialist for internal medicine no. 2, paragraph 38) |
|  | | | **Doubts about time point for measurements** | Built inductively from the data.  If interviewee states that the time point for measurement is not optimal. | “And also that’s early, if I might say so.” (specialist for internal medicine no. 2, paragraph 30) |
|  | | | **Overlapping with other screening instruments** | Built inductively from the data.  If interviewee states that there are other screening instruments that already collect similar information. | “We have to do screening for psycho-oncology, and it would mean we’re having to ask the same questions again. So that’s why I think it’s not really that relevant at the moment.” (specialist for internal medicine no. 2, paragraph 30) |
|  | | | **Enough information about patient already** | Built inductively from the data.  If interviewee states that he or she has enough information about patients — e.g., from the patient history — and that PROs do not provide any additional information. | “Because — just from our point of view now — before treatment, we have very close contact with the patients, we take our time to assess their condition and we think we can recognize the symptoms from a patient history and by seeing the patient during the rounds.” (surgeon no. 1, paragraph 16) |
|  | | | **Unawareness** | Built inductively from the data.  If interviewee states that he or she did not know about using PROs for the individual patients. | “No. Not at all. Not at all, as far as I know. Not even by my colleagues. I did once point it out, but I have a feeling it isn’t established in the everyday routine yet.” (surgeon no. 5, female, paragraph 16) |
|  | **1.2. Potential clinical usage** | | | Category that structures subunits; built from interview guide | Quotes were assigned to smallest subunits in this category |
|  | | **1.2.1. Potential example uses of PROs** | | Built from interview guide.  Category that structures subunits that contain information about how interviewees would use PROs in routine. |  |
|  | | | **Added information** | Built inductively from the data.  If interviewee states that PROs may provide advantages for routine procedures. | “And the questionnaire definitely also includes questions I wouldn’t necessarily ask routinely, so it definitely brings in aspects that might otherwise get lost.” (surgeon no. 2, paragraph 16) |
|  | | | **Preparation for patient consultations** | Built inductively from the data.  If interviewee states that PROs might be used for communication with patients. | “It also answers — and this is important of course when we’re meeting up with the psycho-oncologists and internal-medicine colleagues in oncology, who also use pancreas questionnaires and all that sort of thing to assess quality of life — it will definitely have an influence on conversations with patients, with the patient information process before the operation, so to speak, because I’ll know how some of them feel afterward.” (surgeon no. 3, paragraph 20) |
|  | | | **Screening instrument** | Built inductively from the data.  If interviewee states that PROs might be used to identify patients’ needs and problems. | “In the end, if we used it, I think we’d be able to recognize more quickly which patients need something from us.” (surgeon no. 5, paragraph 22) |
|  | | | **Treatment planning** | Built inductively from the data.  If interviewee states that PROs might be relevant for treatment planning. | “I can imagine using this, or even having it included in treatment planning.” (surgeon no. 1, paragraph 24) |
|  | | | **Feedback to the clinician about therapy** | Built inductively from the data.  If interviewee states that PROs might be useful for evaluating treatment options in general. | "Well, it answered my surgical questions about the long-term outcome.” (surgeon no .3, paragraph 20) |
|  | | | **Monitoring course of disease** | Built inductively from the data.  If interviewee states that PROs might be relevant for seeing the patients’ individual courses. | “And it’s also exciting for them to evaluate what became of our patients and how they got on.” (specialist for internal medicine no. 1, paragraph 16) |
|  | | **1.2.2. Facilitators** | | Built from interview guide.  Category that structures subunits that contain information about facilitating factors for PRO usage. | Quotes are assigned to subunits in this category |
|  | | | **Access** | Built inductively from the data.  If interviewee states her/his opinion about how access to the PROs should be provided. | “Well, anything that’s directly in the computer is better.” (surgeon no. 2, paragraph 38) |
|  | | | **Time point for measurement** | Built inductively from the data.  If interviewee mentions a time point for PRO measurements that would be of interest to him/her. | “When the patients are back home, of course, and everything has settled down. I think then the whole thing becomes even more important again. Because, as I said, in the first 10 or 14 days between, during, and immediately after an operation like that — I’d say until rehabilitation is finished. But here it’s still a ‘worst case’ situation for lots of patients. And then of course it’s difficult to discuss things like that with them.” (nurse no. 3, paragraph 10) |
|  | | | **Coordination** | Built inductively from the data.  If interviewee states a need for coordination of PRO management. | “... You just have to implement some sort of system for what happens with the questionnaire after that. I think that’s the bigger problem …” (surgeon no. 5, paragraph 24) |
| **2. PRO reports** | | | | In the second part of the interview, different presentation styles (Supplementary Material III) for PRO reports were shown to the interviewees so they could be discussed. The following codes contain aspects that were mentioned during this second part of the interview. | Quotes are assigned to subunits in this category |
|  | | **2.1. Overall aspects relative to PRO reports** | | Category that structures subunits; built from interview guide; relevant general aspects that were mentioned on the presentation of PRO results. | Quotes were assigned to subunits in this category |
|  | | | **Development over time** | Built inductively from the data.  If interviewee states that it is important to see the measurement results from different time points. | “Exactly, that shows a course then. That’s very important then.” (specialist for internal medicine no. 1, paragraph 28) |
|  | | | **Problems with interpreting PROs** | Built inductively from the data.  If interviewee states difficulties in interpreting the results due to varying reading directions in the EORTC scales for functioning and symptoms. | “Yes, as I said, to start with it took quite a lot of getting used to, because you jump between functions and symptoms and then it goes back and forth a bit. You first have to get used to it.” specialist for internal medicine no. 1, paragraph 22) |
|  | | | **Structure** | Built inductively from the data.  If interviewee mentions how the different scores should be arranged. | “Well, I think it’s good that it’s on one sheet.” (surgeon no. 4, paragraph 62) |
|  | | | **Reference values/ cut-offs** | Built inductively from the data.  If interviewee gives his/her opinion about reference values. | “Well, I think cut-offs are a bit difficult with questions like this, so I think I’d rather … The thing is, the people are answering it on a scale from one point to another. I think I’d rather look at the questions. I think reference values are a bit difficult here.” (specialist for internal medicine no. 2, paragraph 63) |
|  | | | **Selection of scores** | Built inductively from the data.  If interviewee gives his/her opinion about which scores should be presented (e.g., only notable scores or every score). | “Or perhaps you could differentiate it and say you can make two buttons, with one showing only unusual features for a quick glimpse, or another where I can see the overall analysis of the questionnaire before and after for each patient. That would be great.” (surgeon no. 3, paragraph 46) |
|  | | **2.2. Evaluation of presentation styles** | | Category built from the interview guide; contains aspects regarding the particular presentation styles for PRO reports (Supplementary Material III) that were shown to the interviewees. | Quotes are assigned to subunits in this category |
|  | | | **Colored markings** | Built inductively from the data.  If interviewee gives his/her opinion about colored markings in the presentation styles. | "Yes, I think that’s not bad. Exactly. Especially this thing with the traffic light system, because it shows me at a glance: where exactly do I need to look?” (surgeon no. 5, paragraph 44) |
|  | | | **Line chart** | Built from the interview guide.  If interviewee gives his/her opinion about curve charts for PRO presentation. | “I think this [curve chart] is quite good for the individual patient, because for me it’s just the clearest — is it going down, up, or down again — so you can see at a glance that someone has a postoperative problem, you know, and then again.” (surgeon no. 4, paragraph 56) |
|  | | | **Bar chart** | Built from the interview guide.  If interviewee gives his/her opinion about bar charts for PRO presentation. | “I always associate bar charts with other things.” (surgeon no. 4, paragraph 56) |
|  | | | **Tabular presentation** | Built from the interview guide.  If interviewee gives his/her opinion about tabular presentation of PRO results. | “And actually I think tables nowadays, with scores written up like this, I think that’s not so good for me visually. But that’s because I’m certainly a person who learns visually and also thinks visually.” (surgeon no. 4, paragraph 56) |
