# Supplementary material for: Could existing infrastructure for using patient‐reported outcomes as quality measures also be used for individual care in patients with colorectal cancer?
Source: BMC Health Serv Res. 2021 May 11;21:448. doi: 10.1186/s12913-021-06457-6 (PMC8111716; doi:10.1186/s12913-021-06457-6)

Title: Could existing infrastructure for using patient-reported outcomes as quality measures in several health systems also be used for individual care in patients with colorectal cancer? Clinician perceptions of potential inhibiting and facilitating factors

**Authors**

1. Corresponding author:

Clara Breidenbach
German Cancer Society
Kuno-Fischer-Straße 8, 14057 Berlin, Germany
[breidenbach@krebsgesellschaft.de](mailto:breidenbach@krebsgesellschaft.de); +49 30 322 932 934

1. Christoph Kowalski
   German Cancer Society

Kuno-Fischer-Straße 8, 14057 Berlin, Germany
[kowalski@krebsgesellschaft.de](mailto:kowalski@krebsgesellschaft.de); +49 30 322 932 947

1. Simone Wesselmann
   German Cancer Society

Kuno-Fischer-Straße 8, 14057 Berlin, Germany
[wesselmann@krebsgesellschaft.de](mailto:wesselmann@krebsgesellschaft.de); +49 30 322 932 990

1. Nora Tabea Sibert

German Cancer Society
Kuno-Fischer-Straße 8, 14057 Berlin, Germany
[sibert@krebsgesellschaft.de](mailto:sibert@krebsgesellschaft.de); +49 30 322 932 968

**Additional File 3:** **Presentation Styles PRO Reports**

1. Example for line chart (physical functioning)

*with color-scheme/cut-off points without color-scheme/cut-off points*


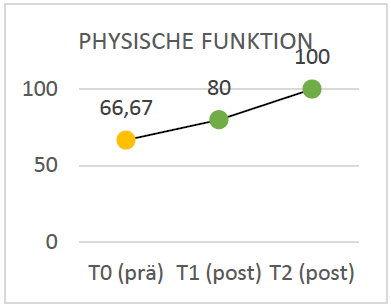

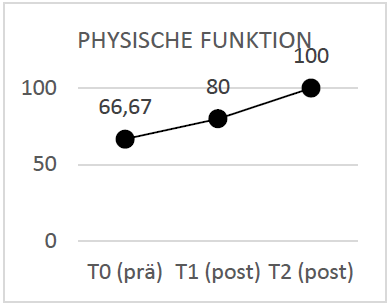


1. Example for bar chart (physical functioning)

*with color-scheme/ cut-off points without color-scheme/ cut-off points*


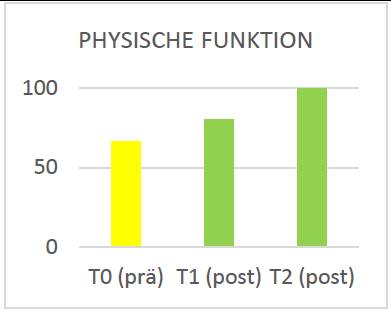

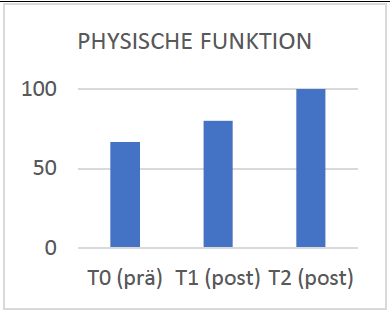


1. Example for tabular presentation (physical functioning)

*with highlighting/ cut-off points*
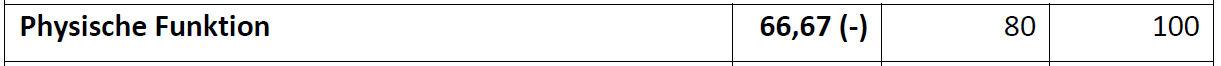


*without highlighting/ cut-off points*


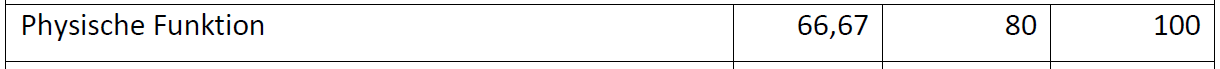

Supplement: Supplementary file 3 — Additional file 3. Presentation Styles PRO Reports [file 12913_2021_6457_MOESM3_ESM.docx]
